# A new species of the *Rhinella margaritifera* species group (Anura, Bufonidae) from the montane forest of the Selva Central, Peru

**DOI:** 10.3897/zookeys.371.6580

**Published:** 2014-01-17

**Authors:** Jiří Moravec, Edgar Lehr, Juan Carlos Cusi, Jesús H. Córdova, Václav Gvoždík

**Affiliations:** 1 Department of Zoology, National Museum, 19300 Praha 9, Czech Republic; 2 Department of Biology, Illinois Wesleyan University, Bloomington, IL 61701, USA; 3 Departamento de Herpetología, Museo de Historia Natural, Universidad Nacional Mayor de San Marcos. Av. Arenales 1256, Lince, Lima 14, Perú

**Keywords:** Amphibia, *Rhinella yunga*, new species, *Rhinella festae*, new species group, Andes

## Abstract

We describe a new species of the bufonid toad genus *Rhinella* from transition montane forest of the buffer zones of the Yanachaga-Chemillén National Park and the Pui Pui Protected Forest (eastern slopes of Andes, Selva Central, Peru). The new species belongs to the *Rhinella margaritifera* species group (confirmed by mtDNA data) and differs from all its members by the absence of tympanic membrane and tympanic annulus. It is characterized by medium size (SVL 57.5–65.5 mm, n = 5), moderately developed cranial crests, absence of neural crest of vertebrae, absence of bone protrusion at angle of jaw, presence of lateral rows of enlarged tubercles, and absence of subgular vocal sac and vocal slits in males. In addition, based on the molecular phylogenetic analyses of selected *Rhinella* species we propose the monophylum containing *R. chavin*, *R. festae*, *R. macrorhina*, *R. manu*, *R. nesiotes*, *R. rostrata*, and *R. yanachaga* as a new species group under the name *Rhinella festae* species group.

## Abstract (es)

Se describe una nueva especie de sapo Bufonidae del género *Rhinella* presente en los bosques montanos transicionales de la zona de amortiguamiento del Parque Nacional Yanachaga Chemillén y el Bosque de Protección Pui Pui (Vertiente Oriental de los Andes, Selva Central, Perú). La nueva especie pertenece al grupo de especies *Rhinella margaritifera* (confirmado con datos de DNAmt) y difiere de todos sus miembros por la ausencia de membrana timpánica y anillo timpánico. Se caracteriza por su tamaño mediano (SVL 57.5–65.5 mm), crestas craneales moderadamente desarrolladas, ausencia de una cresta neural, ausencia de protuberancia ósea en el ángulo mandibular, presencia de filas laterales de tubérculos agrandados, y ausencia de saco vocal subgular y hendiduras vocales en los machos. Además, basado en los análisis filogenéticos moleculares de especies seleccionadas de *Rhinella* se propone la monofilia que contiene a *R. chavin*, *R. festae*, *R. macrorhina*, *R. manu*, *R. nesiotes*, *R. rostrata* y *R. yanachaga* como un nuevo grupo de especies bajo el nombre de grupo de especies *Rhinella festae*.

## Introduction

Neotropical toads of the *Rhinella margaritifera* species group (formerly *Bufo margaritifer* or *Bufo typhonius* species group/complex) are represented by several members of anuran communities inhabiting floor of tropical humid rainforest of South America. These medium-sized (SVL ca. 31–81 mm; e.g., Hoogmoed 1986, Caldwell 1991) ground dwelling toads show obvious adaptations to the life on leaf litter of primary and secondary forests. Their cryptic coloration resembles dark partly decomposed fallen leaves (“dead-leaf pattern”). Effect of this coloration is multiplied by body outline disruptive function of elevated and laterally widely expanded cranial crests, bone protrusions at angle of jaws and neural crests of vertebrae of some species (often sexually dimorphic), which also serve as direct antipredator mechanism. Similar overall external body constitution and typical “dead-leaf” color pattern on one side and substantial intra- and interpopulational variation in the individual morphological characteristics on the other has shown a limited value of external morphology for proper understanding of taxonomy within the *Rhinella margaritifera* species group. Therefore, unusually high cryptic species diversity has been expected within this species group. Reliability of this premise has been confirmed by modern studies based on non-morphological research methods (see Fouquet et al. 2007a).

Currently, 16 species of the *Rhinella margaritifera* species group are recognized (Frost 2013, Lavilla et al. 2013): *Rhinella acutirostris* (Spix, 1824); *Rhinella alata* (Thominot, 1884); *Rhinella castaneotica* (Caldwell, 1991); *Rhinella dapsilis* (Myers & Carvalho, 1945); *Rhinella hoogmoedi* Caramaschi & Pombal, 2006; *Rhinella lescurei* Fouquet, Gaucher, Blanc & Velez-Rodriguez, 2007; *Rhinella magnussoni* Lima, Menin & Araújo, 2007; *Rhinella margaritifera* (Laurenti, 1768); *Rhinella martyi* Fouquet, Gaucher, Blanc & Velez-Rodriguez, 2007; *Rhinella ocellata* (Günther, 1858), *Rhinella paraguayensis* Ávila, Pansonato & Strüssmann, 2010; *Rhinella proboscidea* (Spix, 1824), *Rhinella roqueana* (Melin, 1841); *Rhinella sclerocephala* (Mijares-Urrutia & Arends-R, 2001); *Rhinella scitula* (Caramaschi & Niemeyer, 2003); and *Rhinella stanlaii* (Lötters & Köhler, 2000). Out of these 16 species, six occur in western part of Amazonia (*Rhinella acutirostris*, *Rhinella castaneotica*, *Rhinella dapsilis*, *Rhinella margaritifera*, *Rhinella proboscidea*, and *Rhinella roqueana*) and only one species (*Rhinella stanlaii*) is reported from the humid montane rainforest of the Amazonian versant of the Andes in central Bolivia (elevation range 1600–2000 m a.s.l.; Lötters and Köhler 2000).

During herpetological surveys in the Yanachaga-Chemillén National Park (Cordillera Yanachaga; see Lehr et al. 2012) and Pui Pui Protected Forest (Bosque de Protección Pui Pui) in central Peru, we discovered an apparently unnamed species of *Rhinella margaritifera* species group living in montane forest in the buffer zones of both protected areas. This contribution is aimed to its formal description.

## Materials and methods

### Morphological characters

Collected specimens were fixed in 96% ethanol and stored in 70% ethanol. Measurements are given in millimetres (mm) and were taken by the senior author to the nearest 0.1 mm using a dissecting microscope and electronic digital callipers. Notes on color in life were taken from field notes and color photographs. Webbing formula follows the standards of Myers and Duellman (1982), whereas all other terminology is that of Duellman (1970) and Duellman and Lehr (2009). Format of the description follows the standards of Duellman and Mendelson (1995) and Köhler and Lötters (1999). Measurement abbreviations used throughout the text are: SVL, snout–vent length; TL, tibia length; FL, foot length (distance from proximal margin of inner metatarsal tubercle to tip of Toe IV); HL, head length (from angle of jaw to tip of snout); HW, head width (at level of angle of jaw); ED, horizontal eye diameter; IOD, interorbital distance; EW, upper eyelid width; IND, internarial distance; E–N, eye-nostril distance (straight line distance between anterior corner of orbit and posterior margin of external nares). Fingers and toes are numbered preaxially to postaxially from I–IV respectively I–V. We determined comparative lengths of Toes III and V by adpressing both toes against Toe IV; lengths of Fingers I and II were determined by adpressing the fingers against each other. Condition of the tympanum was assessed by visual examination under microscope. Specimens were sexed externally based on the presence or absence of nuptial pads and internally based on the type of gonads. Photographs taken in the field by JM were used for descriptions of color in life. As locality traits we used GPS coordinates collected in the field based on WGS84 datum. Specimens were deposited in the herpetological collections of the Field Museum, Chicago (FNHM), Museo de Historia Natural, Universidad Nacional Mayor de San Marcos (MUSM) in Lima, Peru, and the National Museum Prague, Czech Republic (NMP). The referred material is registered in the Illinois Wesleyan University, Bloomington, USA (IWU). For specimens examined, see Appendix.

### Molecular phylogenetic analysis

*Species selection*: We included three specimens of the putative new species from the *Rhinella margaritifera* species group from the montane forests of the Cordillera Yanachaga (Selva Central, Peru) and, for comparison, several specimens from the same species group from different lowland localities in the Peruvian and Bolivian Amazonia tentatively identified as *Rhinella* cf. *margaritifera* and *Rhinella* cf. *castaneotica*. We also tested distinctiveness and phylogenetic position of sympatric *Rhinella yanachaga* Lehr, Pramuk, Hedges & Córdova, 2007 and *Rhinella* cf. *leptoscelis* (Boulenger, 1912) collected in the same area of the Cordillera Yanachaga, both from the *Rhinella veraguensis* species group (Lehr et al. 2007, Padial et al. 2009), i.e., a presumed closely-related outgroup (cf. Pramuk 2006). As a definite outgroup we employed a sample of *Rhinella poeppigii* (Tschudi, 1845) from a distant species group of the *Rhinella marina* species group (Pramuk 2006; Maciel et al. 2010). Our dataset was further supplemented by sequences from GenBank to include representatives of all earlier published genetic lineages of the *Rhinella margaritifera* species group (Pramuk 2006; Fouquet et al. 2007a, 2012a, b; Jansen et al. 2011). In addition, some other closely related species outside the *Rhinella margaritifera* species group were also included following phylogenies from Pramuk (2006), Chaparro et al. (2007), Van Bocxlaer et al. (2010), and Pyron and Wiens (2011) [*Rhinella amboroensis* (Harvey & Smith, 1993), *Rhinella chavin* (Lehr, Köhler, Aguilar & Ponce, 2001), *Rhinella nesiotes* (Duellman & Toft, 1979), and *Rhinella veraguensis* (Schmidt, 1857), all presumably from the *Rhinella veraguensis* species group (cf. Padial et al. 2006; Pramuk 2006), and *Rhinella festae* (Peracca, 1904) sequences from GenBank (Frost et al. 2006; Pramuk 2006)]. The resulting dataset was composed of 43 samples with 32 samples from the *Rhinella margaritifera* species group.

*Laboratory procedure*: The genomic DNA was extracted from tissues stored in 96% ethanol. The mitochondrial 16S rRNA gene, which is widely used in the amphibian DNA barcoding (e.g., Vences et al. 2012), was targeted using the primers 16SL1 and 16SH1 adapted or directly taken from Palumbi et al. (1991). For more details, PCR conditions and sequencing see Moravec et al. (2009). The gained sequences have been deposited in GenBank (KF992143–KF992154).

*Computational analysis*: Maximum likelihood (ML) and Bayesian inference (BI) approaches were applied to construct phylogenetic trees to infer evolutionary positions of our samples in the context of published phylogenies of closely related groups of South American toads (cf. Pramuk 2006; Fouquet et al. 2007a). The DNA multiple sequence alignment was carried out in MAFFT v7.1 (Katoh and Standley 2013) producing a 415 bp-long alignment. Ambiguously aligned positions were eliminated by Gblocks v0.91b under options for a less stringent selection (Castresana 2000) producing a final alignment of 400 bp. The software jModelTest v2.1.4 (Darriba et al. 2012) using the PhyML algorithm (Guindon and Gascuel 2003) was used to find the best-fitting model of nucleotide evolution, which was under the Akaike information criterion the GTR+I+G model. The ML analysis was performed in PhyML v3.0 (Guindon et al. 2010) using the best option of a combination of the nearest neighbour interchange and the subtree pruning and regrafting algorithm of tree improvement, and with optimization of the topology and branch lengths. The branch support was assessed by 1000 bootstrap pseudoreplicates. The BI analysis was run in MrBayes v3.2.2 (Ronquist et al. 2012) with two runs and four chains in each run for 6 × 10^6^ generations, sampling every 100th generation. Appropriate sampling was justified by examining the stationarity of log-likelihood scores against the generation time using Tracer v1.5 (Rambaut and Drummond 2009; all parameters had effective sample size > 200), and convergence between the two simultaneous runs was confirmed by the convergence diagnostics of the average standard deviation of split frequencies and the potential scale reduction factor values. From the sampled trees, 25% were discarded as a burn-in and a 50% majority-rule consensus tree was produced from the remaining post burn-in trees. The posterior probabilities (pp) were calculated as the frequency of samples recovering any particular clade. Clades supported with pp values ≥ 0.95 and ML bootstrap values ≥ 70% were considered highly supported (Huelsenbeck and Rannala 2004). Genetic uncorrected *p*-distances were calculated in PAUP* (Swofford 2003).

## Results

### Phylogenetic analysis and systematics

**Figure 1.**
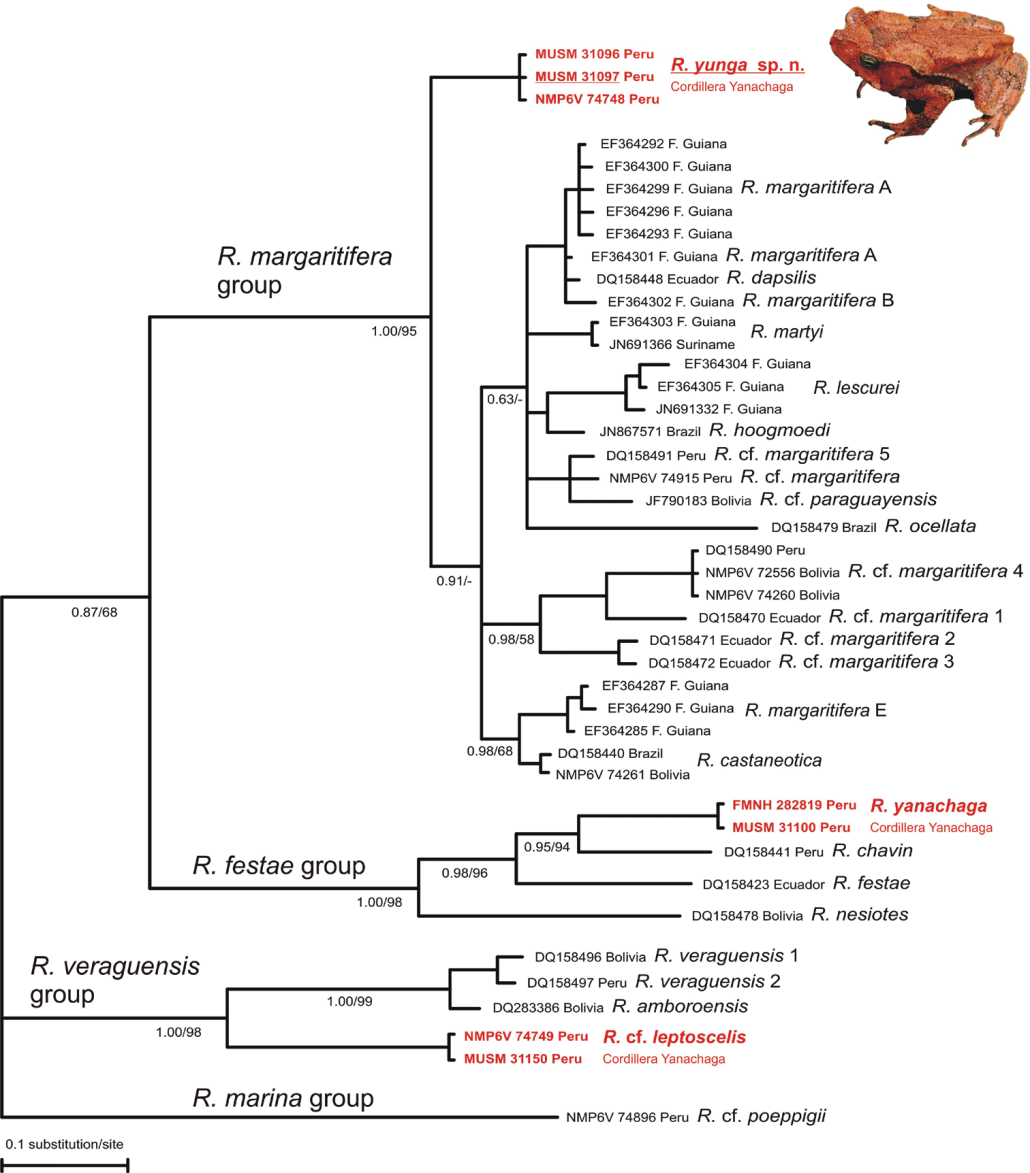
The Bayesian phylogeny of South American *Rhinella* toads with a focus on the *Rhinella margaritifera* group and closely related species. Numbers below the branches correspond to the Bayesian posterior probabilities and ML bootstrap values. Nodes were collapsed if appeared in less than 50% of the post burn-in tree samples. In red the three toad species collected in the Cordillera Yanachaga mountains, Selva Central, Peru, representing members of three different species groups.

The observed morphological differences of the *Rhinella margaritifera* species group specimens from the buffer zone of the Yanachaga-Chemillén National Park were supported by results of the molecular phylogenetic analyses. Both phylogenetic methodological approaches (ML and BI) resulted in essentially same trees (BI tree in Fig. 1). Their common character is a clear division into three well-supported main clades beside the outgroup lineage of the *Rhinella marina* species group (*Rhinella* cf. *poeppigii*). The three species of toads collected in the Cordillera Yanachaga mountains are represented in all three main clades. *Rhinella* cf. *leptoscelis* forms a clade with *Rhinella veraguensis* and *Rhinella amboroensis* (Bayesian pp 1.00/ML bootstrap 98), i.e., the *Rhinella veraguensis* clade. The recently described *Rhinella yanachaga* shows up as a sister species of *Rhinella chavin* and both are members of a well-supported clade (1.00/98) together with *Rhinella festae* and *Rhinella nesiotes* outside the *Rhinella veraguensis* clade. The third species from the Cordillera Yanachaga, the putative new species, is positioned with a high support in the *Rhinella margaritifera* group (1.00/95). This species appears as an independent lineage sister to all other studied (lowland) members of the species group. However, there was low statistical support for this topology similarly like general low supports for the branching inside the group keeping the phylogenetic arrangement within the *Rhinella margaritifera* species group an open question. Genetic distances (*16S*) of the species of the *Rhinella margaritifera* group from the Cordillera Yanachaga from the other taxa of the group were 3.3% (*Rhinella castaneotica*) – 6.0% (*Rhinella* cf. *margaritifera* 1) with the average distance 4.9%.

#### Rhinella yunga sp. n.

http://zoobank.org/36EB4C22-3BCB-4371-90AA-8F5CE2E6B571

http://species-id.net/wiki/Rhinella_yunga

##### Holotype

**Figure 2.**
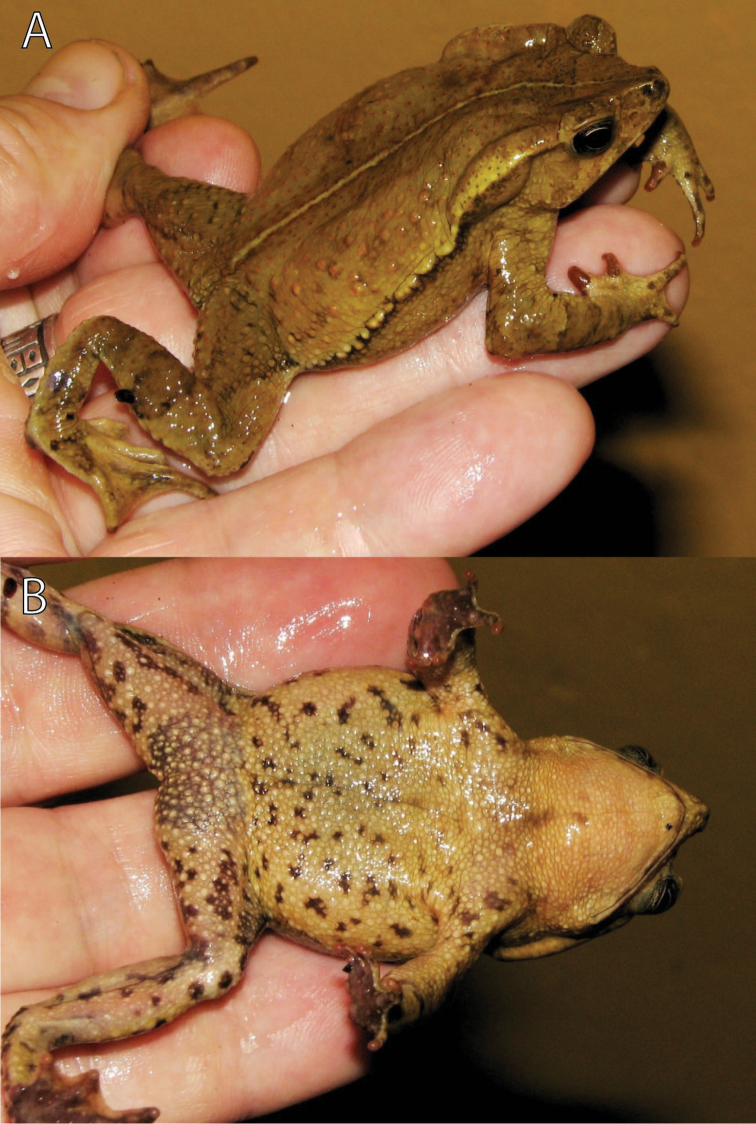
Holotype of *Rhinella yunga* sp. n. (MUSM 31097) in life, (**A**) laterodorsal, and (**B**) ventral views. Photographs by J. Moravec.

**Figure 3.**
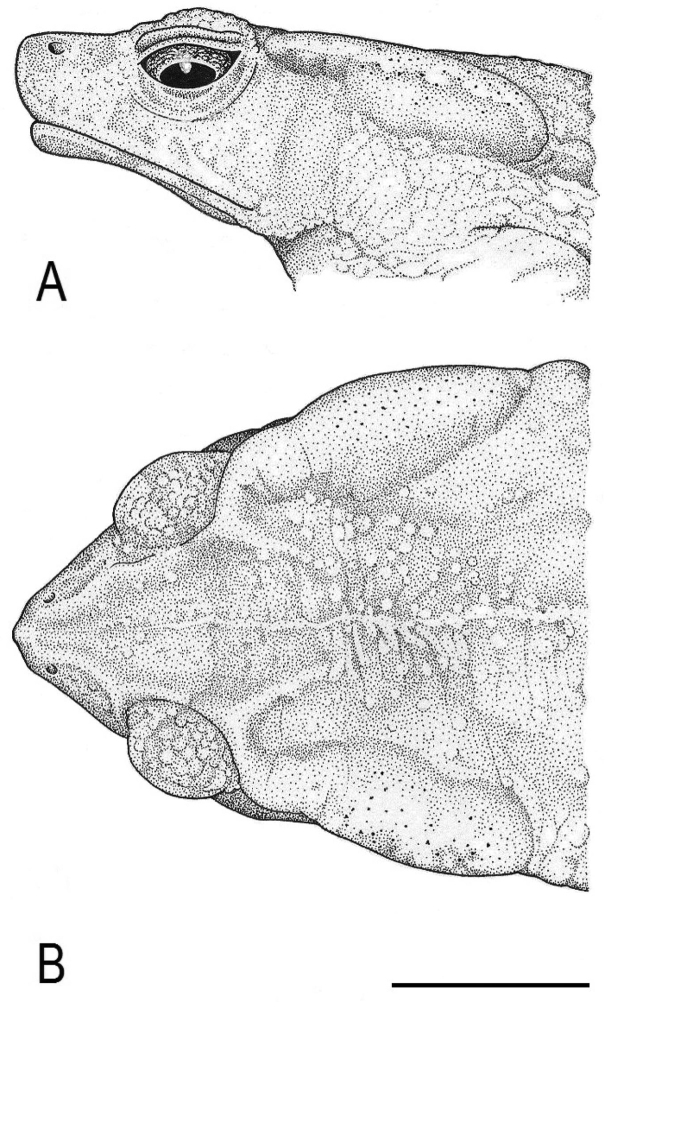
Holotype of *Rhinella yunga* sp. n. (MUSM 31097), (**A**) lateral, and (**B**) dorsal views of head. Scale bar equals 10 mm. Drawings by J. Moravec.

**Figure 4.**
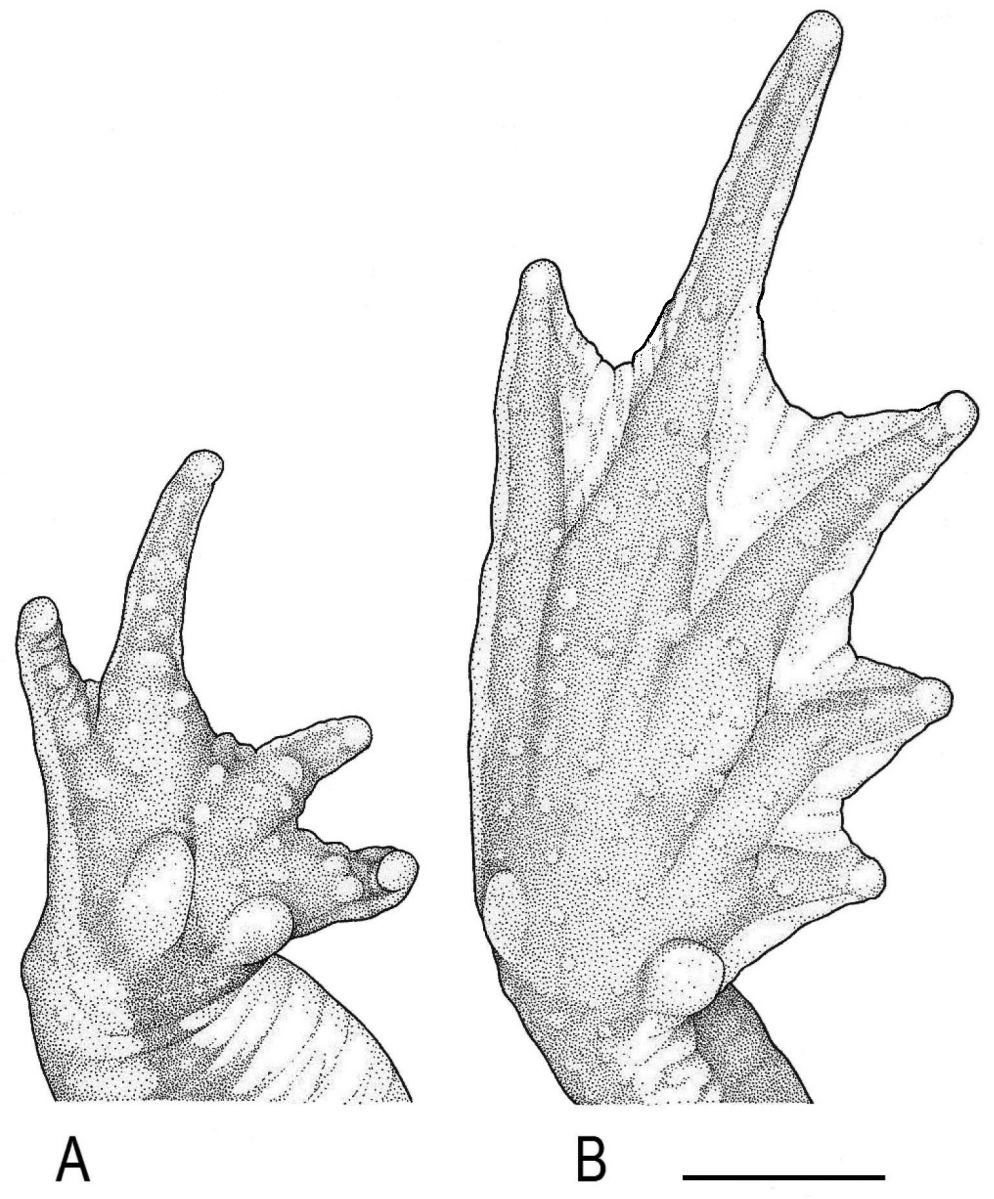
Holotype of *Rhinella yunga* sp. n. (MUSM 31097), (**A**) palmar, and (**B**) plantar views of right hand and foot. Scale bar equals 5 mm. Drawings by J. Moravec.

(Figs 2–4). MUSM 31097 (GenBank *16S rRNA*: KF992151), adult male, collected at Quebrada San Alberto (ca. 10°34'S, 75°23'W) at 1950 m a.s.l., in the buffer zone of the Yanachaga-Chemillén National Park (Sector San Alberto), Distrito de Oxapampa, Provincia de Oxapampa, Región Pasco, Peru, on 15 January 2012 at 18:40h by Edgar Lehr, Jiří Moravec, and Juan Carlos Cusi.

##### Paratypes.

**Figure 5.**
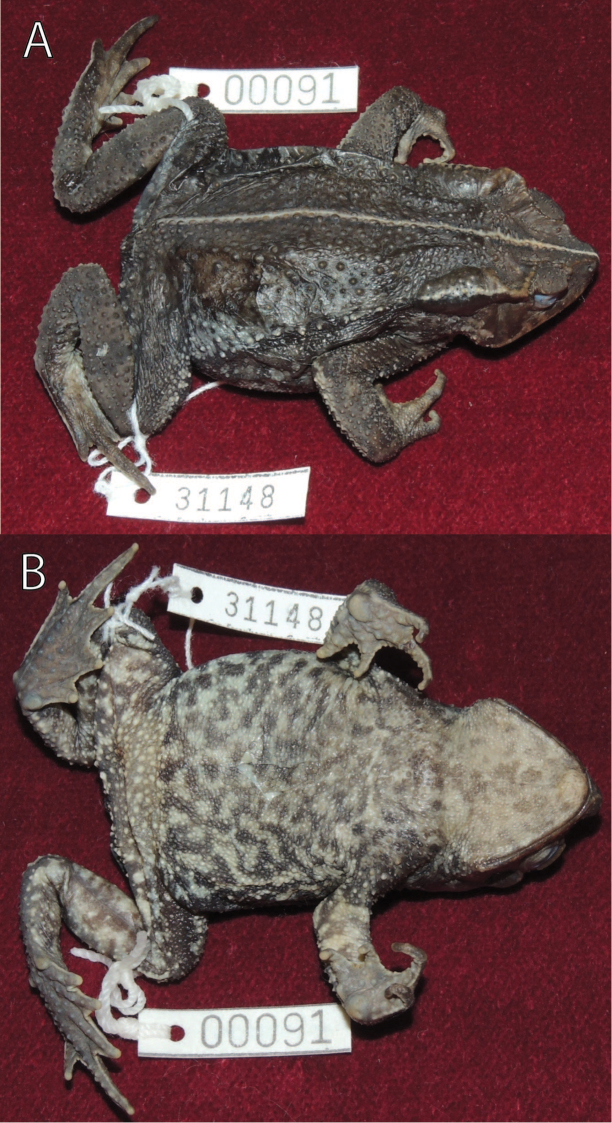
Female paratype of *Rhinella yunga* sp. n. (MUSM 31148) in alcohol, (**A**) lateral, and (**B**) dorsal views. Photographs by J. Moravec.

MUSM 31096, NMP6V 74748 (GenBank *16S rRNA*: KF992150, KF992152), two adult males, collected with the holotype; MUSM 31148 (Fig. 5), an adult female, same locality as holotype, collected on 3 February 2012 at 18:25h by Edgar Lehr, Jiří Moravec, and Juan Carlos Cusi.

##### Referred specimens.

**Figure 6.**
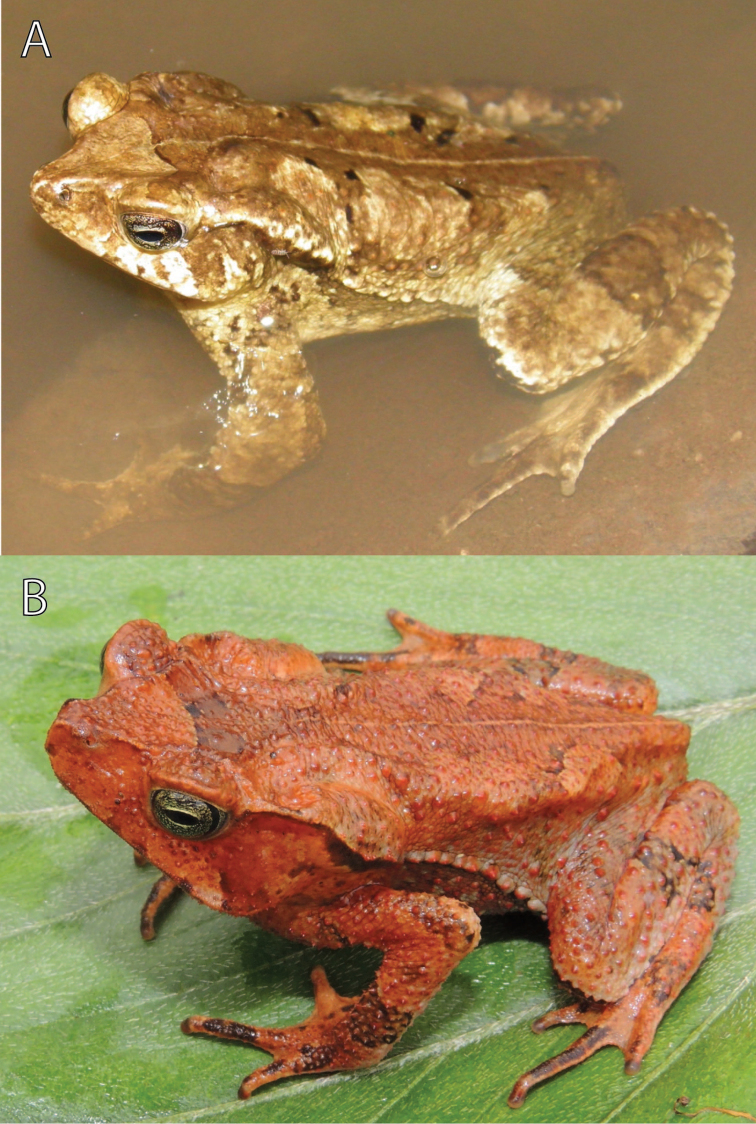
Referred specimens of *Rhinella yunga* sp. n., (**A**) adult male (NMP6F 28) in water, ca. 5 km W of Oxapampa, and (**B**) adult female (IWU 236) from the area of Rio Huatziroki. Photographs by J. Moravec.

NMP6F 28 (photovoucher), adult male (Fig. 6A), observed on the left bank of the Rio Huancabamba, ca. 5 km W of Oxapampa (10°36'S, 75°30'W) at ca. 1885 m a.s.l. on 5 February 2012 by Edgar Lehr, Jiří Moravec, and Juan Carlos Cusi; IWU 236, an adult female (Fig. 6B), collected in the area of Rio Huatziroki (ca. 11°07'04.2"S, 75°12'05.6"W) at 2075 m a.s.l., in the buffer zone of the Protected Forest Pui Pui, Provincia Chanchamayo, Región Junín, Peru, on 13 June 2013 by Rudolf von May and Juan Carlos Cusi; IWU 235 and IWU 273, subadult specimens, collected in the area of Rio Huatziroki (ca. 11°07'04.2"S, 75°12'05.6"W, and 11°07'40.6"S, 75°11'15.7"W), at 1915 and 2230 m a.s.l., in the buffer zone of the Protected Forest Pui Pui, Provincia Chanchamayo, Región Junín, Peru, on 13 and 16 June 2013 by Edgar Lehr, Jiří Moravec, Rudolf von May, and Juan Carlos Cusi.

##### Diagnosis.

A medium-sized species of the *Rhinella margaritifera* speciesgroup characterized by the presence of cephalic crests, distinct parotoid glands, lateral row of tubercles, dorsal “dead-leaf” pattern, and mtDNA data (see Ávila et al. 2010 and Fig. 1). The new species can be distinguished by the following combination of characters: (1) medium size SVL 57.5–59.5 mm in males (n = 3), 53.5–65.5 mm in females (n = 2); (2) snout slightly pointed in dorsal view, protruding beyond the margin of lip, rounded above and curved posteroventrally in profile; (3) nostrils protuberant, directed dorsolaterally, anterior part exceeding anterior margin of lower jaw; (4) canthal, supraorbital and supratympanic crests continuous, slightly elevated in males, distinctly elevated in female; supratympanic crest moderately expanded dorsolaterally in female; (5) tympanic membrane and tympanic annulus absent; (6) bone protrusion at angle of jaw absent; (7) neural crest of vertebrae absent; (8) parotoid glands elongate, elliptical to subtriangular, slightly protruding laterally, incorporated into lateral row of tubercles; (9) lateral row of tubercles present; tubercles rounded to subconical in males, conical in female; (10) skin on dorsum smooth with scattered flat tubercles in male, tubercles conical in female; (11) skin on dorsal surfaces of limbs smooth with scattered low tubercles in males, spinulose in female; (12) first finger slightly longer than the second in males, both fingers equal in length in single female; (13) palmar tubercle large, ovoid, two to four times size of subtriangular thenar tubercle; (14) inner metatarsal tubercle ovoid, protruding distally, ca. two times size of outer rounded to ovoid subconical metatarsal tubercle; (15) modal webbing on foot: I 0^1/4^–0^1/4^ II 0^1/4^–2^–^ III 1^–^–3 IV 3–0^1/4^V in males and I 1^–^–2 II 0^1/3^–2^1/2^ III 1^–^–3^1/4^ IV 3^1/4^–1^–^ V in the single female; (16) subarticular tubercles prominent, round to oval; supernumerary tubercles round, one half to same size of former; (17) subgular vocal sac and vocal slits absent, and nuptial excrescences present in males; (18) dorsum light yellowish tan to reddish-brown, with irregular brown, dark brown or back markings; whitish or pale yellow middorsal stripe present; venter light orange-tan with irregular dark brown spots, iris silvery greenish with irregular black mottling.

##### Comparisons.

Morphologically, *Rhinella yunga* differs from all members of the *Rhinella margaritifera* species group by the absence of tympanum. From the currently recognized species of the *Rhinella margaritifera* species group occurring in the area of the eastern slopes of the Andes and lowland Western Amazonia, the new species can also be distinguished by following combinations of characters: from *Rhinella acutirostris* by larger size, absence of bone protrusion at angle of jaw and by coloration (*Rhinella acutirostris*: SVL up to 47 mm in males [35.3 mm in adult male holotype] and 57 mm in females, weak bone protrusion at angle of jaw, belly cream in holotype; Spix 1824, Hoogmoed 1986, Lötters and Köhler 2000); from *Rhinella castaneotica* by larger size, presence of lateral rows of enlarged tubercles and absence of bone protrusion at angle of jaw (*Rhinella castaneoti* ca: SVL 30.9–36.8 mm in males, 33.8–42.6 mm in females, lateral rows of enlarged tubercles absent, weak bone protrusion at angle of jaw present; Caldwell 1991, Köhler and Lötters 1999, Ávila et al. 2010); from *Rhinella dapsilis* by smaller size, absence of fleshy process in the snout, and tuberculate to spinulose skin (*Rhinella dapsilis*: SVL 77 mm in female holotype, snout developed in a fleshy proboscis, skin smooth; Myers and Carvalho 1945, Rodrígues and Duellman 1994, Fouquet et al. 2007b); from *Rhinella margaritifera* by less developed cranial crests, absence of neural crest of vertebrae, and absence of bone protrusion at angle of jaw (*Rhinella margaritifera*: supraorbital and supratympanic crests hypertrophied, bone protrusion at angle of jaw and vertebral apophyses present; Hoogmoed 1986, Fouquet et al. 2007b, Ávila et al. 2010, type specimen ZISP 257.1 in Milto and Barabanov 2011: fig. 17, Lavilla et al. 2013); from *Rhinella proboscidea* by less prominent and less pointed snout, distinct parotid glands, tuberculate to spinulose skin, and presence of lateral row of tubercles (*Rhinella proboscidea*: snout distinctly prominent and pointed, parotid glands indistinct, smooth skin, lateral row of tubercles absent; Spix 1824, Hoogmoed 1986); from *Rhinella roqueana* by smaller size, less expanded supratympanic crest, absence of bone protrusion at angle of jaw, absence of neural crest of vertebrae, and presence of lateral row of tubercles (*Rhinella roqueana*: SVL up to 72 mm in males and 81 mm in females, supratympanic crest large, bone protrusion at angle of jaw well developed, crest of vertebrae present, lateral row of tubercles absent; Hoogmoed 1986); from *Rhinella stanlaii* by larger size, absence of bone protrusion at angle of jaw, and by coloration (*Rhinella stanlaii*: SVL 39.1–54.1 mm in males and 57.2–59.4 mm in females, well developed bone protrusion at angle of jaw, ventral colors brown and cream; Lötters and Köhler 2000).

##### Description of holotype.

Adult male; body robust; SVL 59.5 mm; head wider than long; snout slightly pointed in dorsal view, protruding beyond the margin of lip, rounded above and curved posteroventrally in profile; nostrils protuberant, directed dorsolaterally, anterior part exceeding anterior margin of lower jaw; canthus rostralis concave in lateral view, rounded in profile; loreal region barely concave, horizontal eye diameter larger than distance between nostril and anterior corner of eye; temporal region curved caudomedially; tympanic membrane and tympanic annulus absent; canthal, supraorbital, and supratympanic crests continuous; canthal crest low, barely distinct; supraorbital and supratympanic crests slightly elevated, supratympanic crest slightly expanded laterally, not exceeding markedly outer edge of upper eyelid; bone protrusion at angle of jaw absent; neural crest of vertebrae absent; parotoid glands well developed, elongate, subtriangular, slightly protruding laterally; lateral row of rounded to subconical tubercles from posterior margin of parotoid gland to groin (the first tubercle separated from the gland). Skin on dorsal and lateral surfaces smooth with scattered low to conical tubercles lacking keratinized tips; cranial crests and parotoid glands smooth; loreal and temporal areas smooth with sporadic inconspicuous flat tubercles; upper eyelids with prominent round tubercles; skin on throat and belly coarsely areolate to warty. Forelimb hypertrophied; relative length of fingers II < IV < I < III; palmar tubercle prominent, ovoid; thenar tubercle conspicuously prominent, subtriangular, about one third size of the palmar tubercle; subarticular tubercles large, prominent, distal subarticular tubercle under Finger III bifid; supernumerary tubercles numerous, about half size or less of subarticular tubercles; basal webbing between fingers; nuptial excrescences present on thenar tubercle, dorsal and lateral surfaces of Fingers I–II and inner lateral surface of finger III. Foot longer than tibia; relative length of toes I < II < V < III < IV; inner metatarsal tubercle ovoid, protruding distally; outer metatarsal tubercle ovoid, subconical, about half the size of inner metatarsal tubercle; subarticular tubercles prominent, round to oval; supernumerary tubercles round, about half to same size of subarticular tubercles; toes with moderate webbing, webbing formula I 0^1/4^–0^1/4^ II 0^1/4^–2^–^ III 1^–^–3 IV 3–0^1/4^V; lateral fringes broad; tips of digits terminating in indistinct discs. Tongue elongate, attached to anterior part of mouth floor; choanae small, oval; vocal slits absent; subgular vocal sac absent.

Measurements of holotype provided in Table 1.

**Table 1.** Measurements (mm) of the holotype and the paratypes of *Rhinella yunga* sp. n. (see text for abbreviations).

Measurements	Holotype	Paratypes		
	MUSM 31097	MUSM 31096	NMP6V 74748	MUSM 31148
**Sex**	Male	Male	Male	Female
**SVL**	59.5	57.5	58.0	65.5
**TL**	22.9	23.0	23.8	23.4
**FL**	25.7	24.4	24.8	25.0
**HL**	17.9	17.3	17.4	19.9
**HW**	20.0	19.6	20.0	22.7
**ED**	6.2	6.1	6.1	6.0
**IOD**	7.1	6.9	6.7	7.7
**EW**	4.2	4.0	4.0	4.1
**IND**	2.8	2.9	2.9	3.5
**E-N**	4.2	4.0	3.9	4.0
**PL**	14.2	12.3	11.6	9.9
**PW**	5.7	5.9	5.9	6.0

##### Coloration of holotype in alcohol.

Dorsal surfaces of head, body, and limbs light grey with slightly darker irregular markings forming very inconspicuous “dead-leaf pattern” from between eyes to cloacal region; whitish grey middorsal stripe from snout to cloaca. Middle area of shank, tibia, and tarsus with obscure dark grey spots forming one transverse bar on flexed leg. Dark grey transverse bar on forearm. Ground color of lateral side of head and body light grey. Two inconspicuous oblique darker grey bars below eye, one darker grey bar in temporal area from posterior edge of eye to angle of jaw. Dorsal side of parotoid glands and lateral row of tubercles light grey, sharply contrasting with dark grey to black longitudinal stripe leading from posterior margin of orbit, along lateral side of parotoid gland and below the lateral row of tubercles. Throat, belly, and ventral surfaces of legs whitish with irregular dark grey spots.

##### Coloration of holotype in life.

General pattern same as in alcohol. Ground color yellow tan dorsally, orange tan ventrally; larger scattered dorsal tubercles light orange.

Iris silvery greenish with irregular black mottling.

##### Variation.

For variation in measurements see Table 1. The male paratypes are similar to the holotype in body form and coloration. An uncollected male observed ca. 5 km W of Oxapampa (Fig. 6A) differed in more contrast “dead leaf pattern”. The female paratype (Fig. 5) is larger than the holotype, is more tuberculate (larger tubercles possess keratinized tips), has distinctly elevated cranial crests with supratympanic crest moderately expanded dorsolaterally, and differs in less developed webbing (see Diagnosis). The overall dorsal coloration of the female paratype is darker and the dark spots on the throat and belly are denser than in the holotype. The morphological characters of the three referred specimens from the buffer zone of the Pui Pui Protected Forest correspond, in general, to those of the type series. The referred adult female (IWU 236; Fig. 6B) measured 53.5 mm in SVL and its coloration (in life) is intense reddish-brown.

##### Etymology.

The specific name *yunga* is derived from the Quechua expression *yungas* meaning “warm valley”, which is widely used for an ecoregion of montane rainforests covering the eastern Andean slopes of Peru and Bolivia. The name is used as a noun in apposition and refers to the general habitat of the new species.

##### Distribution, ecology, and threat status.

**Figure 7.**
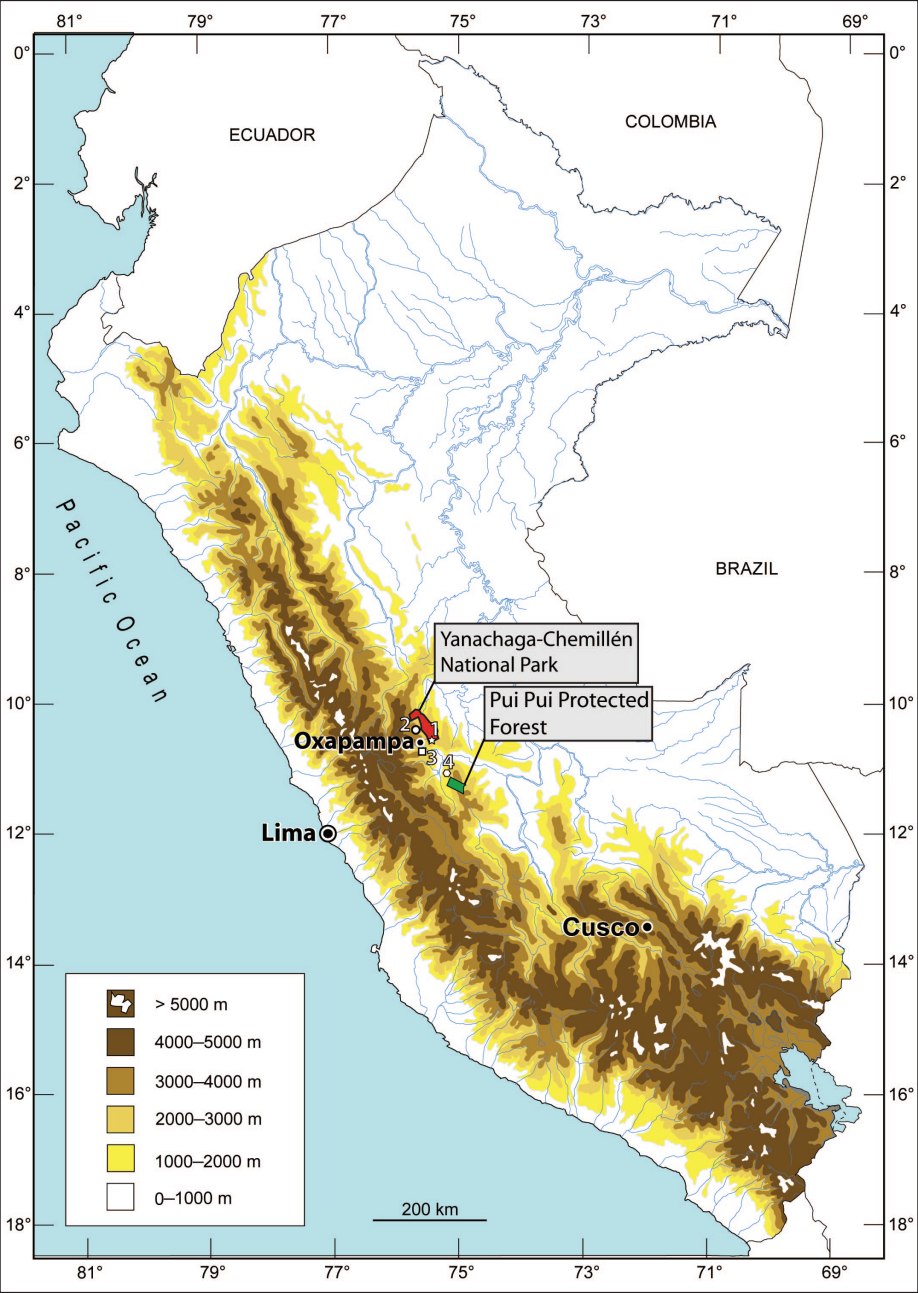
Schematic map of central and southern Peru showing known distribution of *Rhinella yunga* sp. n. **1** type locality **2** Quebrada Yanachaga valley at the settlement Prosoya (elevation ca. 1800 m. a.s.l.) **3** Rio Huancabamba (ca. 5 km W of Oxapampa, ca. 1885 m a.s.l.) **4** Rio Huatziroki (elevation 1915–2230 m a.s.l.) lying in the buffer zone of the Pui Pui Protected Forest ca. 60 km straight southeast of the type locality. Map by E. Lehr.

Besides from its type locality, *Rhinella yunga* is also known from the area on the left bank of the Rio Huancabamba (ca. 5 km W of Oxapampa, ca. 1885 m a.s.l.), from Quebrada Yanachaga valley at the settlement Prosoya (10°25.118'S, 75°31.126'W, ca. 1800 m. a.s.l.) and from the area of Rio Huatziroki (elevation 1915–2230 m a.s.l.) lying in the buffer zone of the Pui Pui Protected Forest ca. 60 km straight southeast of the type locality (see the referred material; Fig. 7). To date, *Rhinella yunga* is known from an altitudinal range 1800–2230 m a.s.l., which represents a contact belt between the transitional montane forest (“Bosque de transición”, 1000–2000 m a.s.l.) and montane cloud forest (“Bosque de neblina”, 2000–3400 m a.s.l.; altitudinal zonation adopted from Milanovich et al. 2006). It is likely that *Rhinella yunga* is distributed in a wider area of montane forests in the Peruvian regions Pasco and Junín (Selva Central).

**Figure 8.**
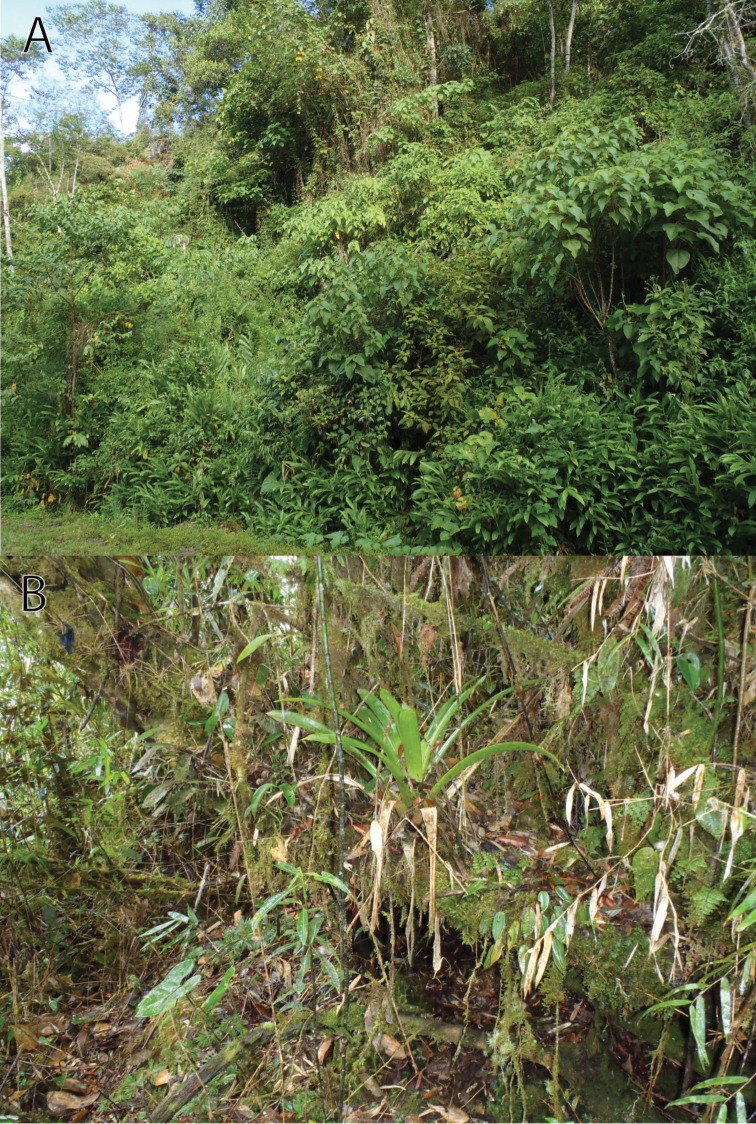
Habitat of *Rhinella yunga* sp. n., (**A**) a road margin at the type locality, and (**B**) closed cloud forest in the area of Rio Huatziroki (ca. 2200 m a.s.l.). Photographs by J. Moravec.

All collected and observed specimens were in breeding condition. The female paratype contained numerous small pigmented oviductal eggs. At the type locality, the males were sitting in shallow water of small temporal water bodies along a narrow unpaved road (Fig. 8A). The males entered the water in the late afternoon. The female paratype was collected on the ground in close vicinity of breeding puddles at night. In the vicinity of Huancabamba-Prosoya (Programa Social Yanachaga, former Hacienda Yanachaga) one adult male was observed (not collected) in a small artificial pond at night. The three referred specimens from the area of Rio Huatziroki were found in low dense forest covering a sharp montane ridge (Fig. 8B). Tadpole and call are unknown. Observed sympatric anurans include *Rhinella* cf. *leptoscelis* (MUSM 31150, NMP6V 74749), *Hypsiboas aguilari* Lehr, Faivovich & Jungfer, 2010 (MUSM 31147), *Pristimantis* cf. *bipunctatus* (Duellman & Hedges, 2005), and *Pristimantis* sp. We classify *Rhinella yunga* as “Data Deficient” according to the IUCN red list criteria and categories (IUCN Standards and Petitions Subcommittee 2013) based on the limited information on its geographic range.

## Discussion

It appears that large number of still unnamed cryptic species remains hidden under some nominal species of the *Rhinella margaritifera* species group (e.g., Pramuk 2006; Fouquet et al. 2007a; Lavilla et al. 2013). The main reason of this fact is a limited use of traditional morphological methods for a proper delimitation of many of still undescribed taxa. Nevertheless, the newly recognized *Rhinella yunga* is a rare exception. It differs from all members of the *Rhinella margaritifera* species group by an absence of an external tympanum. Lack of tympanum in combination with other morphological features shows that *Rhinella yunga* is a separate, morphologically well-defined species. In addition, this finding is supported also by genetic data. Despite the very preliminary character of the obtained phylogeny of the selected species of the genus *Rhinella*, it is evident that *Rhinella yunga* represents a separate lineage within the radiation of the *Rhinella margaritifera* species group (Fig. 1). Its current position in the phylogenetic tree seems to be sister to all other studied members of the species group (lowland), suggesting a possible basal split between the montane Andean and lowland Amazonian taxa. However, the statistical support for this scenario is presently rather low and a further testing with more complete taxon sampling is necessary. *Rhinella yunga* is distributed in the area of montane forest at the western limit of the range of the *Rhinella margaritifera* species group, where it occurs syntopically with morphologically and genetically clearly divergent members of the *Rhinella veraguensis* species group (*Rhinella* cf. *leptoscelis*; Fig. 1). The recently described *Rhinella yanachaga* was expected to be a part of the *Rhinella veraguensis* group (Lehr et al. 2007), however this species shows up in a well-supported clade outside the *Rhinella veraguensis* clade (Fig. 1). A similar topology of such an outside clade has been already shown in former studies (however without *Rhinella yanachaga*; Pramuk 2006; Chaparro et al. 2007; Van Bocxlaer et al. 2010; Pyron and Wiens 2011). Therefore, based on this phylogenetic arrangement, which makes the *Rhinella veraguensis* species group (sensu Pramuk 2006; Padial et al. 2006) paraphyletic, we propose the clade containing *Rhinella yanachaga* as a new species group under the name *Rhinella festae* species group. According to the currently available data (Pramuk 2006; Chaparro et al. 2007; Van Bocxlaer et al. 2010; Pyron and Wiens 2011), the *Rhinella festae* species group contains the following species: *Rhinella chavin*, *Rhinella festae*, *Rhinella macrorhina* (Trueb, 1971), *Rhinella manu* Chaparro, Pramuk & Gluesenkamp, 2007, *Rhinella nesiotes*, *Rhinella rostrata* (Noble, 1920), and *Rhinella yanachaga*.

## Supplementary Material

XML Treatment for Rhinella yunga

## Appendix

### Specimens examined

*Rhinella castaneotica*: BOLIVIA: Pando, Federico Roman: Santa Crucito, ca. 150 m a.s.l.: NMP6V 74261 (GenBank *16S rRNA*: KF992144).

*Rhinella* cf. *margaritifera*: PERU: Ucayali: Pucallpa: 17.4 km S of Masisea, ca. 160 m a.s.l.: NMP6V 74915 (GenBank *16S rRNA*: KF992143).

*Rhinella* cf. *margaritifera*: BOLIVIA: Pando: Nicolas Suarez: Bioceanica, ca. 250 m a.s.l.: NMP6V 72556 (GenBank *16S rRNA*: KF992146).

*Rhinella* cf. *margaritifera*: BOLIVIA: Pando: Manuripi: San Antonio, ca. 270 m a.s.l.: NMP6V 74260 (GenBank *16S rRNA*: KF992145).

*Rhinella yanachaga*: PERU: Pasco: Oxapampa: Quebrada Yanachada, 2900 m a.s.l.: FNHM 282819 (GenBank *16S rRNA*: KF992148), MUSM 31100 (GenBank *16S rRNA*: KF992149).

*Rhinella* cf. *leptoscelis*: PERU: Pasco: Oxapampa: Quebrada San Alberto, 1950 m a.s.l., NMP6V 74749 (GenBank *16S rRNA*: KF992153).

*Rhinella* cf. *leptoscelis*: PERU: Pasco: Oxapampa: ca 5 km W Oxapampa, ca. 1885 m a.s.l., MUMS 31150 (GenBank *16S rRNA*: KF992154).

*Rhinella* cf. *poeppigii*: PERU: Ucayali: Boquerón Padre Abad, ca. 415 m a.s.l.: NMP6V 74896 (GenBank *16S rRNA*: KF992147).
